# Purpuric Type Drug Eruption Caused by Azithromycin: A Case Report and Literature Review

**DOI:** 10.7759/cureus.54214

**Published:** 2024-02-14

**Authors:** Natsuko Saito-Sasaki, Yu Sawada

**Affiliations:** 1 Dermatology, University of Occupational and Environmental Health, Kitakyushu, JPN

**Keywords:** skin, case report, purpuric type, drug eruption, azithromycin

## Abstract

Azithromycin, an azolide antibiotic with structural and functional similarities to macrolides, possesses distinct features such as its effects persisting for seven days, an extended half-life by administering it once daily for three days, and strong antimicrobial activity. Notably, vomiting and diarrhea are recognized as the primary adverse events related to azithromycin.

In this particular case, we present a unique case describing a purpuric-type drug eruption associated with azithromycin, which represents an uncommon cutaneous manifestation. A 64-year-old female developed a purpuric eruption on her trunk and lower extremities seven days after receiving daily intravenous azithromycin for upper bronchitis. A previous occurrence of punctate purpuric eruption following azithromycin administration was documented in her medical history. The diagnosis of azithromycin-induced skin eruption was confirmed based on the clinical progression and the recurrence of the eruption upon re-administration of the drug. In response to this diagnosis, the patient underwent treatment involving the discontinuation of azithromycin and the application of topical betamethasone butyrate propionate ointment. Remarkably, her eruption significantly improved within two weeks, although residual pigmentation persisted post-treatment. Additionally, we offer a comprehensive review of the literature, examining cases of drug eruptions related to azithromycin.

## Introduction

Azithromycin, an azolide antibiotic, belongs to the class macrolides and shares both structural and functional similarities with this category against bacteria [1]. Its remarkable features, encompassing an extended half-life and robust antimicrobial efficacy, set it apart within the antibiotic spectrum [2]. Notably, azithromycin is known for its prolonged duration of action and potent activity against various microorganisms [3,4].

Despite its clinical effectiveness, azithromycin is not without its drawbacks, as vomiting and diarrhea have been identified as primary adverse effects associated with its administration [5]. In the context of adverse reactions, this report presents a distinctive case - an inaugural instance of a purpuric-type drug eruption associated with azithromycin administration. This represents a unique and uncommon cutaneous manifestation, expanding the spectrum of potential side effects associated with this antibiotic. The purpuric-type drug eruption observed in this case underscores the importance of vigilant monitoring for diverse adverse reactions, even those considered rare, in patients undergoing azithromycin therapy.

Moreover, recognizing the need for a comprehensive understanding of the literature surrounding drug eruptions attributed to azithromycin, this report provides a literature review [6-12]. By synthesizing and analyzing previously published case reports, we aim to contribute valuable insights into the broader landscape of azithromycin-related adverse cutaneous reactions. This comprehensive review not only aids in enhancing our understanding of the drug's safety profile but also serves as a resource for clinicians to evaluate potential risks and benefits in prescribing azithromycin for various clinical scenarios.

## Case presentation

**Figure 1:**
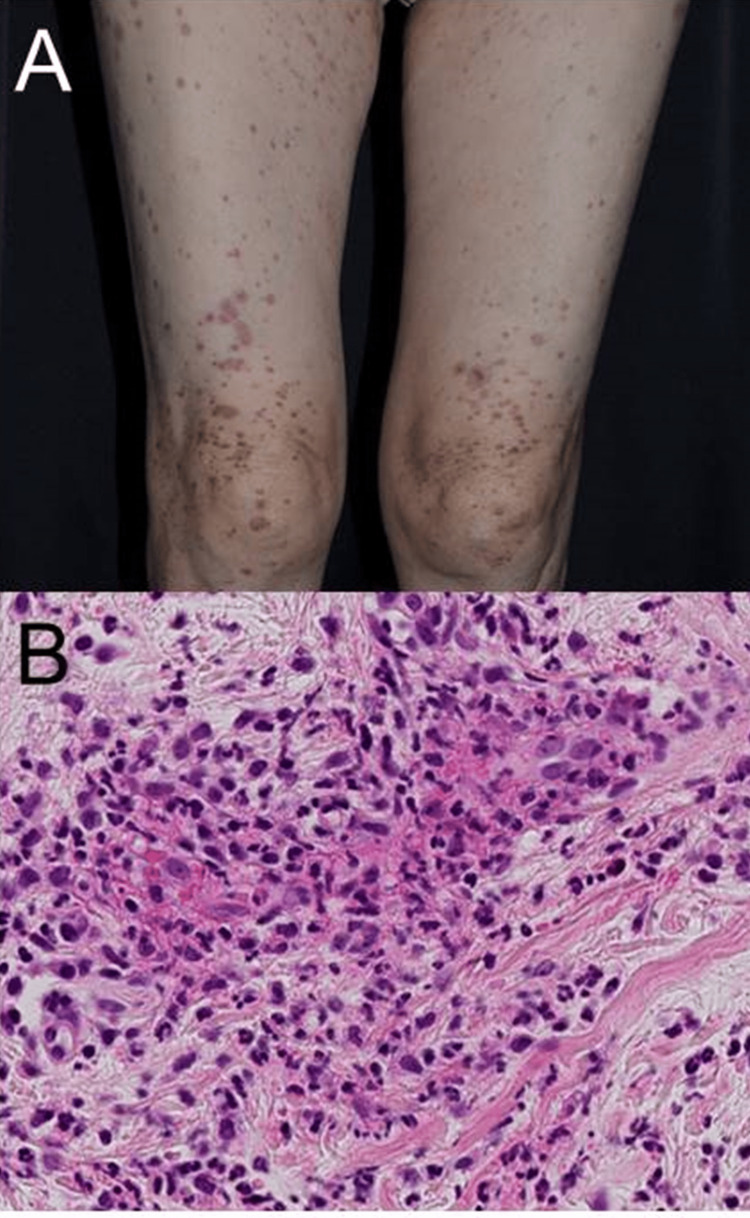
Clinical manifestation and histological analysis (A) Clinical manifestation showing palpable purpuric lesions on her trunk. (B) Histopathology of the skin showing lymphocyte, neutrophil, and eosinophil infiltration in the upper dermis. Nuclear dust and erythrocyte extravasation were also observed.

Seven days after receiving daily intravenous doses of 2 g of azithromycin for upper bronchitis, a 64-year-old woman experienced the development of a purpuric eruption on her trunk and lower extremities, prompting her referral to our department for comprehensive evaluation. She had no other comorbidities or prescribed medications. This skin eruption occurred subsequent to a prior episode of punctate purpuric eruption following azithromycin administration. Upon thorough physical examination, palpable purpuric lesions were identified on her trunk and both legs (Figure 1A). She had no symptoms of arthritis, diarrhea, abdominal pain, and lower leg edema.

Notably, the patient had no pertinent history of autoimmune diseases, including vasculitis, and reported no concurrent use of any other medications. The white blood cell count (WBC) was recorded at 6300/μl, with eosinophils constituting 3.2% (201.6/μl). Liver function tests indicated aspartate aminotransferase (AST) at 13 U/l and alanine aminotransferase (ALT) at 9 U/l. Additionally, the creatinine level was measured at 0.32 mg/dl. The urinalysis revealed no significant alterations. During the initial visit, a skin biopsy taken from a purpuric lesion on her trunk revealed the presence of lymphocytes, neutrophils, and eosinophils infiltrating around vessels. Notable histopathological findings included nuclear dust and erythrocyte extravasation observed in the upper dermis, indicating the presence of vasculitis (Figure 1B). It's noteworthy that there was no deposition of IgA in the vessel wall, denying the possibility of IgA vasculitis.

Drawing upon the clinical course and the reproducibility of the skin eruption following azithromycin administration, a diagnosis of azithromycin-induced eruption was established based on the clinical course and histological analysis [13]. The therapeutic approach involved discontinuing azithromycin and initiating treatment with topical betamethasone butyrate propionate ointment. Within a span of two weeks, significant improvement in the eruption was observed, although some residual pigmentation persisted post-treatment.

## Discussion

Macrolides are considered the safest of all antibiotics as far as an adverse effect is concerned. The mere temporal association and the effect itself point toward a rare adverse event. Various drugs, particularly thiamine propyl disulfide and chlordiazepoxide, have been identified as causing purpuric-type drug eruptions [14]. To our knowledge, this is the first case of a purpuric-type drug eruption attributed to azithromycin. Given the widespread use of azithromycin as an antibiotic, we conducted a review of reported English cases documenting drug eruptions associated with azithromycin to elucidate its characteristics. A total of eight cases, including our own (Table I), have been reported [6-12]. The mean age of this population was 32.0. This might reflect that azithromycin is prescribed in various population ages. The male/female ratio was 1.6. The types of skin eruptions observed were as follows: two cases of Stevens-Johnson syndrome (SJS), two cases of drug reaction with eosinophilia and systemic symptoms syndrome (DRESS), two cases of maculopapular eruptions, one case of toxic epidermal necrolysis (TEN), and one case of purpuric-type eruption. Therefore, azithromycin-related drug eruption showed various clinical characteristics. The average days of the onset of drug eruption following azithromycin administration was 6.9 days. Notably, 62.5% of the cases manifested as severe cutaneous adverse reactions (SJS, DRESS, and TEN).

**Table 1:** Case reports of drug eruption caused by azithromycin DRESS: drug reaction with eosinophilia and systemic symptoms syndrome

Author	Age	Sex	Eruption type	Interval	
Brkljacić N, et al. [6]	62	Female	Stevens-Johnson syndrome	4 days	
Aihara T, et al. [7]	5	Male	Stevens-Johnson syndrome	3 days	
Dakdouki GK, et al. [8]	19	Male	Maculopapular	same day	
Bauer KA, et al. [9]	8	Male	Drug reaction with eosinophilia and systemic symptoms	4 days	
Sriratanaviriyakul N, et al. [10]	44	Male	Drug reaction with eosinophilia and systemic symptoms	7 days	
Trevisi P, et al. [11]	34	Female	Toxic pustuloderma	16 hours	
Schissel DJ, et al. [12]	20	Male	Maculopapular	7 days	
Our case	64	Female	Purpuric eruption	7 days	

## Conclusions

While our case exhibited a relatively mild manifestation of drug eruption, it is crucial to be aware that azithromycin administration could potentially lead to various clinical types of cutaneous drug eruption. Because azithromycin is available for various age populations, our case report succinctly outlines the clinical features of the cutaneous adverse reaction associated with azithromycin, providing valuable insights for potential encounters in clinical scenarios. Because a limited number of cases of drug eruption due to azithromycin has been reported, the results of similar case studies are required to elucidate the clinical characteristics of azithromycin-related cutaneous adverse events.
